# Arecoline Hydrobromate

**Published:** 1903-01

**Authors:** Walter Lincoln Bell

**Affiliations:** Brooklyn, N. Y.


### ARECOLINE HYDROBROMATE.

By Walter Lincoln Bell, D.V.S.,
BROOKLYN, N. Y.

Arecoline is the alkaloid of areca catechu, L. (Betel nut). The hydrobromate is the only form in which it is used. This occurs in white crystals, soluble in water and in alcohol. Prof. Froehner states that as a laxative it is ten times stronger than pilocarpine and fully as powerful as eserine, while its action is equal to about 1000 times its weight of powdered areca nut. On the strength of his experience, he regards grs 1½ (equal to 3¼ oz. tincture areca nut)

as the maximum dose for horses, and grs. 4 as the maximum dose for oxen.

This drug came to me described as an active sialagogue, rapid diluent, and evacuant of the bowels; indicated in acute indigestions (colics), intestinal obstruction and laminitis, also recommended as a tænifuge; contraindicated in nervous colics, pregnancy, and pharyngitis.

Early in the present year my attention was called to this drug by Dr. H. H. Newcomb, who had just returned from South Africa, where he acted as Veterinary Lieutenant for the British Army. He stated that severe cases of acute laminitis were rendered serviceable in three days by the daily hypodermic injection of arecoline for that period. This, to me, seemed almost impossible, but, as Dr. Newcomb's advice had been of much benefit in previous instances, I secured some tablets of the drug, with the determination of giving it an early trial. My first experience was so satisfactory that I continued its use and have adopted it altogether as one of the most important therapeutic factors in treating this form of disease.

Its rapid action as an evacuant induced me to try it on a patient with acute indigestion and tympanites. I found that it assisted very materially in restoring the animal to early use and entire relief of the acute attack. Since then I have used it very frequently in "colics," and have found that it has all the good qualities claimed for eserine and pilocarpine, alone or combined, barium chloride, etc., but as yet have seen none of the dangerous actions—*i. e.*, fatal dyspnœa, rupture of the diaphragm, stomach, or intestines that sometimes follow the use of eserine and pilocarpine.

I have also used arecoline hydrobromate in five cases of azoturia in conjunction with sedatives and diuretics, and have secured that number of successful cures. While I do not think I have found a specific for this unwelcome disease, still the rapid dilution and expulsion of the intestinal contents, the diaphoresis and profuse excretion of saliva, thus calling upon some of the most important organs of elimination, theoretically, and, as it seems in my experience, practically, has been of decided benefit.

In referring to this drug, it seems of much importance to emphasize the rapid and profuse salivation that follows, usually within five minutes of its hypodermic use. The animal opens his mouth and a large quantity of saliva drops out, and from this time the salivation continues; in some cases for an hour, gradually lessening in quantity.

In laminitis I make a daily injection of one to one and a half grains of arecoline dissolved in one drachm of boiled water that has sufficiently cooled for hypodermic use, and usually select a spot on the neck that the mane will cover, injecting it slowly into the subcutaneous tissues. In conjunction with this I remove the shoes, encase the feet in a thick covering of oakum that has soaked in hot water; this is held on by a burlap and cotton bandages which cover the entire dressing. Then, if possible for the horse to stand, keep him standing in hot water purified with sanitas, creolin, or carbolic preparation during the day, leaving small powders of potassium nitrate to be given every four hours day and night to insure attention to the animal at least that often, giving directions that the feet be immersed in hot water every time a powder is given during the night, the dressings to remain on until I remove them. This procedure I have found will render the animal fit for moderate service after the third day in many instances, and not later than the fifth day in any case so far. Previous to using arecoline I could not secure such good results, though the other parts of the treatment were as rigidly insisted upon.

In azoturia I follow the same general rule, using one to one and a half grains of arecoline in hot aqueous solution daily for three injections, combined with sedatives and diuretics.

Acute indigestions ("colics"), whether accompanied by flatulence or not, are greatly benefited by one-half to one grain in one drachm of hot water. This with hot rectal injections of water with a small amount of glycerine added, used through a large sized fountain syringe and giving sedatives such as chloral hydrate, cannabis indica, etc., I have found very successful as far as any one treatment could be in relieving many cases of "colics," and the arecoline seems to exert a very beneficial action where there is considerabe gastric disturbance. Shortly after the onset of salivation the animal passes wind, soon feces are evacuated, and both continue to pass for some time; the animal secures relief, is out of pain, and the acute attack is over. As arecoline adds considerably to the intestinal pain and inconvenience for a short time, I usually administer the sedative previous to injecting the drug, the quantity of sedative governed by the amount of pain I first find the animal in.

In conclusion I would say that I find it best to use hot water for the hypodermic solution, not only for the aseptic properties, but that it makes a quicker and more thorough solution; also to clean the needle as soon as through with it, as otherwise it becomes entirely stopped up. This is especially true if cold water is used.

---

**Secretary Waugh, of the Allegheny County Veterinary Medical Association, reports the society in active work and planning for aggressive work during the winter season.**

